# Abnormal CD4 + T helper (Th) 1 cells and activated memory B cells are associated with type III asymptomatic mixed cryoglobulinemia in HCV infection

**DOI:** 10.1186/s12985-015-0324-2

**Published:** 2015-07-01

**Authors:** Fanyun Kong, Wei Zhang, Bo Feng, Henghui Zhang, Huiying Rao, Jianghua Wang, Xu Cong, Lai Wei

**Affiliations:** Peking University People’s Hospital, Peking University Hepatology Institute, Beijing Key Laboratory of Hepatitis C and Immunotherapy for Liver Diseases, No.11 Xizhimen South Street, Beijing, 100044 China; Department of Pathogenic biology and Laboratory of Infection and Immunology, Xuzhou Medical College, 84 West Huaihai Road, Xuzhou, 221002 Jiangsu Province China

**Keywords:** Hepatitis C, Type III asymptomatic cryoglobulinemia, CD4+ T helper cells, Activated memory B cells

## Abstract

**Background:**

Mixed cryoglobulinemia (MC) in hepatitis C virus (HCV) infection is associated with abnormal immune responses mediated by T cells and B cells, while the relationships of different subsets of CD4 + T helper (Th) cells, B cells and associated cytokines with type III asymptomatic MC in HCV infection are poorly understood.

**Methods:**

Fifty-four chronic hepatitis C (CHC) patients and 23 healthy controls (HCs) were enrolled in the study. Serum cryoglobulins were detected by cryoprecipitation. The types of cryoglobulin were determined by western blot. The phenotypes and frequencies of Th cell and B cell subsets were detected by flow cytometric analysis. The cytokines IFN-γ, IL-4, IL-17, IL-21, IL-22, and TGF-β were measured by enzyme-linked immunosorbent assay.

**Results:**

Twenty-six CHC patients were detected with type III asymptomatic MC. The frequencies of Th2, Th17, follicular helper T (Tfh cells), Th22, and tissue-like B cells were significantly higher in CHC patients compared to HCs, while these cell subsets were not significantly different between CHC patients and HCV-related MC patients. The frequencies of Th1 and activated memory B cells increased in HCV-related MC patients compared to HCs, although the difference between the two cell subsets in CHC patients and HCs was not significant. The frequency of regulatory T cells (Treg cells) was higher in CHC patients than in HCV-related MC patients and HCs. Higher expressions of serum IFN-γ, IL-17, IL-21, and IL-22 were observed in CHC patients than in HCs, but the differences were not significantly different in CHC patients and HCV-related MC patients. The frequency of Th1 cells was associated with activated memory B cells in HCV-related MC patients, and the frequency of Th1 cells and activated memory B cells was closely related to HCV RNA in HCV-related MC patients.

**Conclusions:**

The increased frequencies of Th17 cells, Tfh cells, Th22 cells, Treg cells, cytokines IL-17, IL-21, IL-22, and tissue-like B cells, were related to HCV infection but not type III asymptomatic MC. Higher frequencies of Th1 cells and activated memory B cells were associated with type III asymptomatic MC in HCV infection.

## Background

Chronic hepatitis C virus (HCV) infection is not only responsible for hepatic diseases but is also associated with several extrahepatic manifestations. Mixed cryoglobulinemia (MC), an autoimmune disease characterized by the formation of cold-precipitable cryoglobulin complexes composed of immunoglobulins, is recognized as the most common extrahepatic disease induced by HCV infection [1, 2]. Based on the immunoglobulin composition, MC has two types of cryoglobulins: Type II cryoglobulins consisting of polyclonal IgG and monoclonal IgM with rheumatoid factor (RF) activity, and type III cryoglobulins characterized by polyclonal IgG with polyclonal IgM. In HCV-related MC, the majority of patients have asymptomatic MC, and only 10 –15 % of these patients will develop cryoglobulinemic symptoms, characterized by small vessel vasculitis, glomerulo-nephritis, and neuropathy due to immune complex deposition and activation of the complement cascade in small blood vessels [3, 4].

To date, the involvement of host immune responses mediated by T cells and B cells with MC in chronic hepatitis C (CHC) patients is poorly understood. Several cytokines and chemokines, including IL-6, TNF-α, IL-1β, IFN-γ, MIP-1α, CXCL9, CXCL10, and CXCL11 associated with T helper (Th) 1 immune responses, were found to be involved in HCV-related type II MC [5–13], especially in patients with autoimmune thyroiditis or active vasculitis. Theoretically, agents that selectively neutralize molecules such as CXCL10, might increase patient responsiveness to HCV therapy [14]. Recent evidence also showed a deficiency of CD4 + CD25+ regulatory T (Treg) cells in HCV-related MC patients with active vasculitis [15]. In addition, a highly restricted T cell receptor variable β gene repertoire in liver and peripheral blood was found in HCV-related MC patients, suggesting that the clonally expanded TCR Vβ repertoire was associated with HCV-related MC [16].

MC is considered to be a benign B-cell proliferative disorder. The expansion of IgM + CD27 + CD21-B cells, which express VH1-69 and VH4-34 genes, are present in type II MC patients with HCV infection [17, 18], and effective treatment for HCV-related MC decreased the number of VH1-69 + B cells [19]. In addition, the expansion of immature B cell subsets and activated memory B cells with distinct activated markers such as CD86 and CD71 were noted in HCV-related MC patients [20]. In contrast, naive B cells in MC patients with HCV infection are decreased because they have a higher sensitivity to apoptosis, and treatment of MC with rituximab restored normal B cell compartments [21]. This suggested that B cells play an important role in the pathogenesis of MC with HCV infection.

Th cell subsets such as Th2 cells (IL-4 + CD4 + T cells), Th17 cells (IL-17 + CD4 + T cells), Th22 cells (IL-22 + CD4+ T cells) and Treg cells are associated with HCV infection [22–25]. To our knowledge, no study has evaluated the immune phenotype of Th cell subsets and their relationship with B cells in CHC patients with type III asymptomatic MC. The identification of immunological markers associated with T and B cell immune responses in type III asymptomatic MC may help to improve clinical monitoring and disease management. To characterize the features and relationships of peripheral Th cell and B cell subsets in CHC patients with type III asymptomatic MC, 26 CHC patients with type III asymptomatic MC, 28 CHC patients without MC and 23 sex- and age-matched healthy controls (HCs) were enrolled in the study. We analyzed different immune cell subsets and Th cell cytokines to identify distinct phenotypes associated with type III asymptomatic MC in HCV infection.

## Results

### Increased percentage of Th1 cells in type III asymptomatic MC with HCV infection

**Fig. 1:**
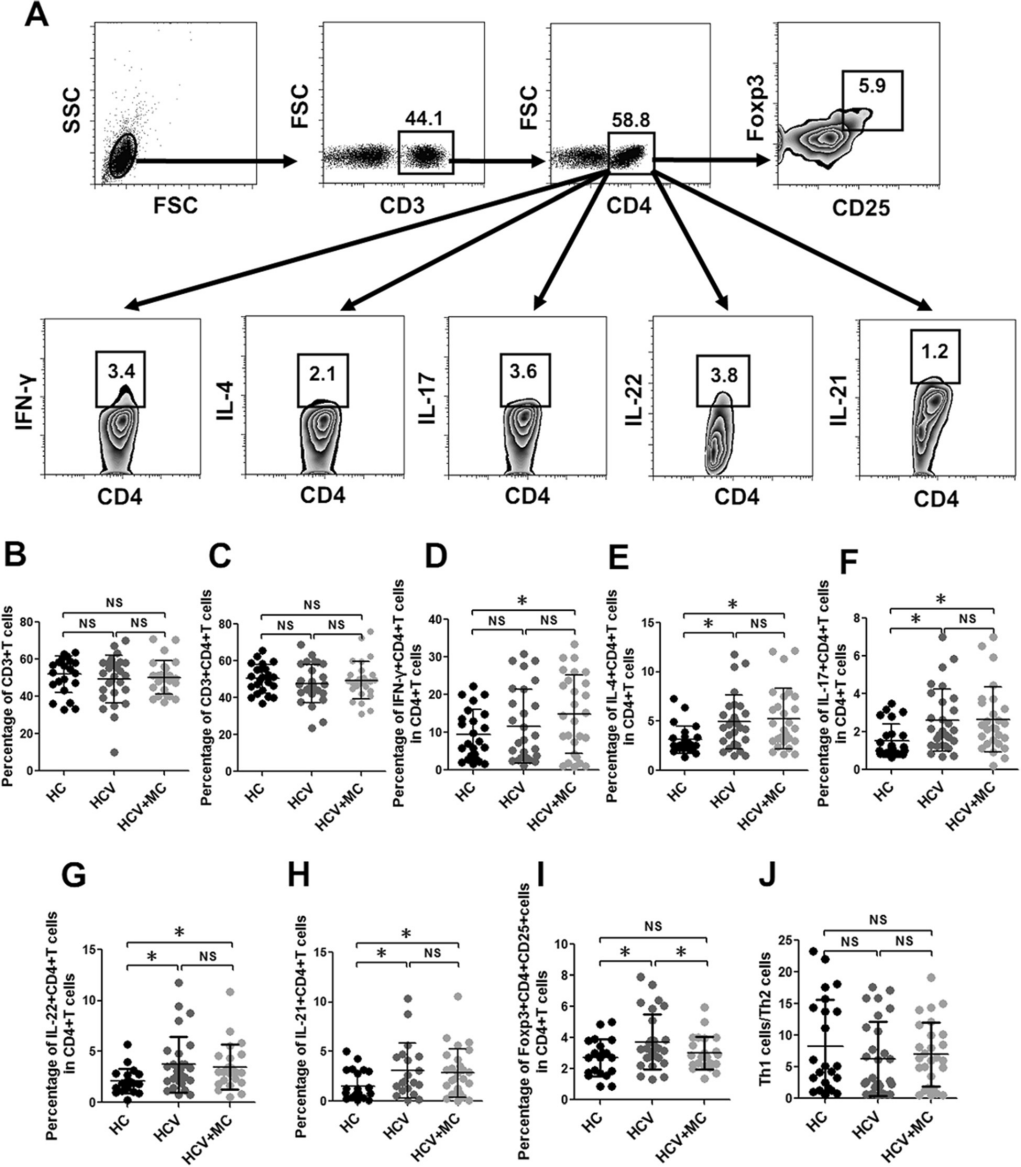
Frequencies of CD4 + T cell subsets in CHC patients and HCV-related asymptomatic MC patients. **a** The gating strategy for T cell subsets by flow cytometric analysis. **b** The frequencies of total T cells. **c** The frequency of CD4 + T cells in total T cells. **d**, **e**, **f g**, **h**, and **i** show the frequencies of IFN-γ + CD4+ T cells, IL-4 + CD4+ T cells, IL-17 + CD4+ T cells, IL-22 + CD4+ T cells, IL-21 + CD4+ T cells, and Treg cells (CD4 + CD25 + Foxp3+ cells) in total CD4+ T cells. **j** Shows the ratio of Th1 cells to Th2 cells. *p <0.05

Among 54 CHC patients, 26 CHC patients were tested positive for type III MC, and none of these MC patients had cryoglobulinemic syndrome (Table 1). To assess the potential role of different subsets of T cells, especially Th cell subsets, in type III asymptomatic MC with HCV infection, total T cells, CD4+ T cells, and CD4+ T cell subsets, including Th1 cells (IFN-γ + CD4+ T cells), Th2 cells, Th17 cells, Th22 cells, and Treg cells (CD4 + CD25 + Foxp3+ cells) were analyzed in CHC patients, HCV-related MC patients, and HCs. We did not note any difference in the frequencies of CD3+ T cells and CD4+ T cells in the three groups (Fig. 1b-c). The frequency of Th1 cells in total CD4+ T cells was significantly higher in MC patients compared with HCs (Fig. 1d). Though a higher frequency of Th1 cells was found in CHC patients, the difference was not significant between CHC patients and HCs. The percentages of Th2 cells, Th17 cells, and Th22 cells in total CD4+ T cells were increased in both CHC patients and HCV-related MC patients compared with HCs (Fig. 1e-g). The frequencies of Th2 cells, Th17 cells, and Th22 cells were not significantly different between CHC patients and HCV-related MC patients, suggesting that higher frequencies of Th2 cells Th17 cells, and Th22 cells were associated with HCV infection but were not related to MC. CD4+ T cells are a main source of IL-21, a cytokine responsible for the activation and differentiation of T cells and B cells [26, 27]. We also measured the frequency of IL-21 + CD4+ T cells in three groups, and found their percentage was increased in CHC patients and HCV-related MC patients (Fig. 1h). However, there was no correlation between the frequency of IL21 + CD4+ T cells and MC. We next detected the frequency of Treg cells in three groups, and found their frequency was higher in CHC patients than in HCV-related MC patients and HCs. However, the frequency of Treg cells was not significantly different between HCV-related MC patients and HCs (Fig. 1i). The ratio of Th1 cells/Th2 cells was also examined in three groups, but there was no significant difference among these groups (Fig. 1j). Taken together, these results indicated that Th2 cells, Th17 cells, Th22 cells, IL21 + CD4+ T cells, and Treg cells were only associated with HCV infection but not MC, while Th1 cells were related to type III MC in HCV infection.

**Table 1:** Baseline characteristics of CHC patients, HCV-related MC patients, and HC

Parameters	CHC	HCV-related MC	HC
Number of subjects	28	26	23
Age (years), mean ± SD	53.6 ± 6.7	55.5 ± 7.1	52.0 ± 8.6
Sex, male/female	16/12	14/12	13/10
ALT, mean ± SD	25.8 ± 25.3	27.5 ± 26.2	23.6 ± 18.1
Genotype (1b)	28	26	—
Type of cryoglobulin	—	III	—
HCV-RNA (log10 IU/mL), mean ± SD	6.81 ± 0.56	6.45 ± 0.94	—

### Increased frequency of activated memory B cells in type III asymptomatic MC with HCV infection

**Fig. 2:**
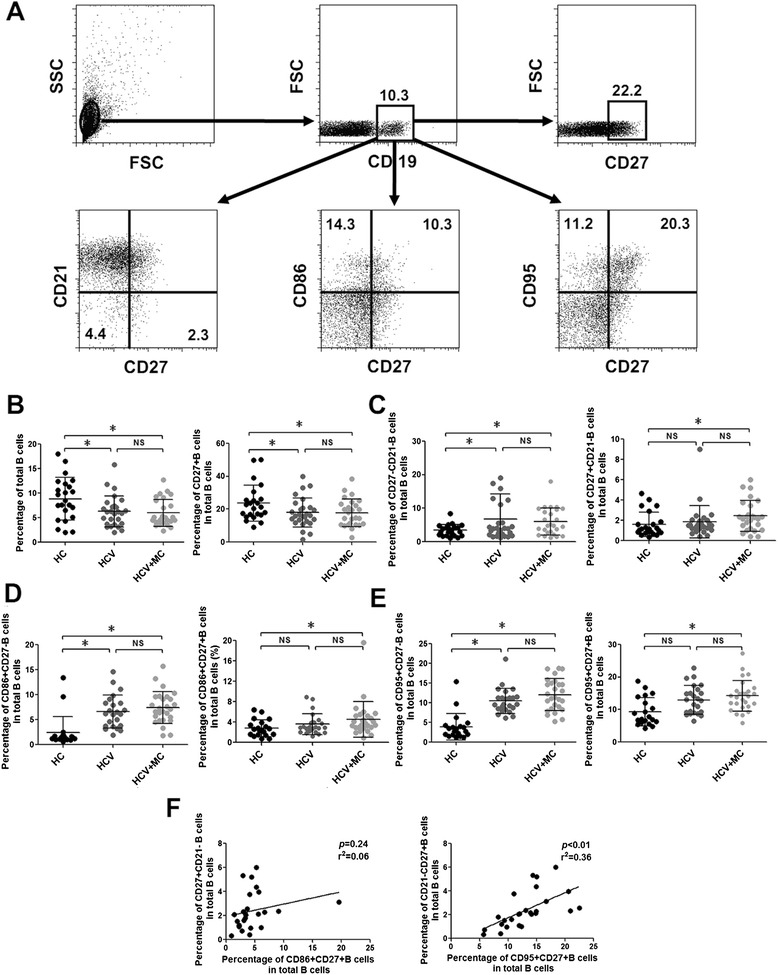
Frequencies of B cell subsets in CHC patients and HCV-related asymptomatic MC patients. **a** The gating strategy for B cell subsets by flow cytometric analysis. **b** The frequencies of total B cells and memory B cells. The frequencies of **c** CD21- B cells in CD27- B cells and CD27+ B cells; **d** CD86+ B cells in CD27- B cells and CD27+ B cells; and **e** CD95+ B cells in CD27- B cells and CD27+ B cells. **f** The relationships between the frequency of CD27 + CD21- B cells, CD86 + CD27 + IgG+ B cells and CD95 + CD27 + IgG+ B cells.*p <0.05

Current studies have reported abnormal B cells in type II MC with HCV infection [17, 18, 20], while few studies have investigated the characteristics of B cells in HCV-related type III asymptomatic MC. As shown in Fig. 2b, the frequencies of total B cells and memory B cells (CD27+ B cells) were decreased in CHC patients. The frequencies of total B cells and memory B cells were comparable between CHC patients and HCV-related MC patients. Measurement of activated B cells, including tissue-like B cells (CD27-CD21- B cells) and activated memory B cells (CD27 + CD21- B cells) in three groups indicated the frequency of tissue-like B cells were increased in both CHC patients and HCV-related MC patients (Fig. 2c), but the difference of percentages of tissue like B cells in CHC patients and HCV-related MC patients was not significant. The percentage of activated memory B cells was only increased in HCV-related CHC patients compared with HCs. Because the increased expression of CD86 and CD95 is associated with B cell activation [28], we detected the expression of CD86 and CD95 in B cell subsets. We found that the increased frequencies of CD86 + CD27- B cells and CD95 + CD27- B cells were comparable in CHC patients and HCV-related MC patients, compared to HCs (Fig. 2d-e). Increased percentages of CD86 + CD27+ B cells and CD95 + CD27+ B cells were observed in HCV-related MC patients but not in CHC patients when compared with HCs. Moreover, in HCV-related MC patients, the frequency of activated memory B cells was positively correlated with CD95 + CD27+ B cells (Fig. 2f). These findings indicate that the increased frequencies of tissue-like B cells are associated with HCV infection, while activated memory B cells with an increase percentage of CD95 + CD27+ B cells is related to type III asymptomatic MC in HCV infection.

### Increased frequency of circulating Tfh cells is associated with HCV infection but not type III asymptomatic MC

**Fig. 3:**
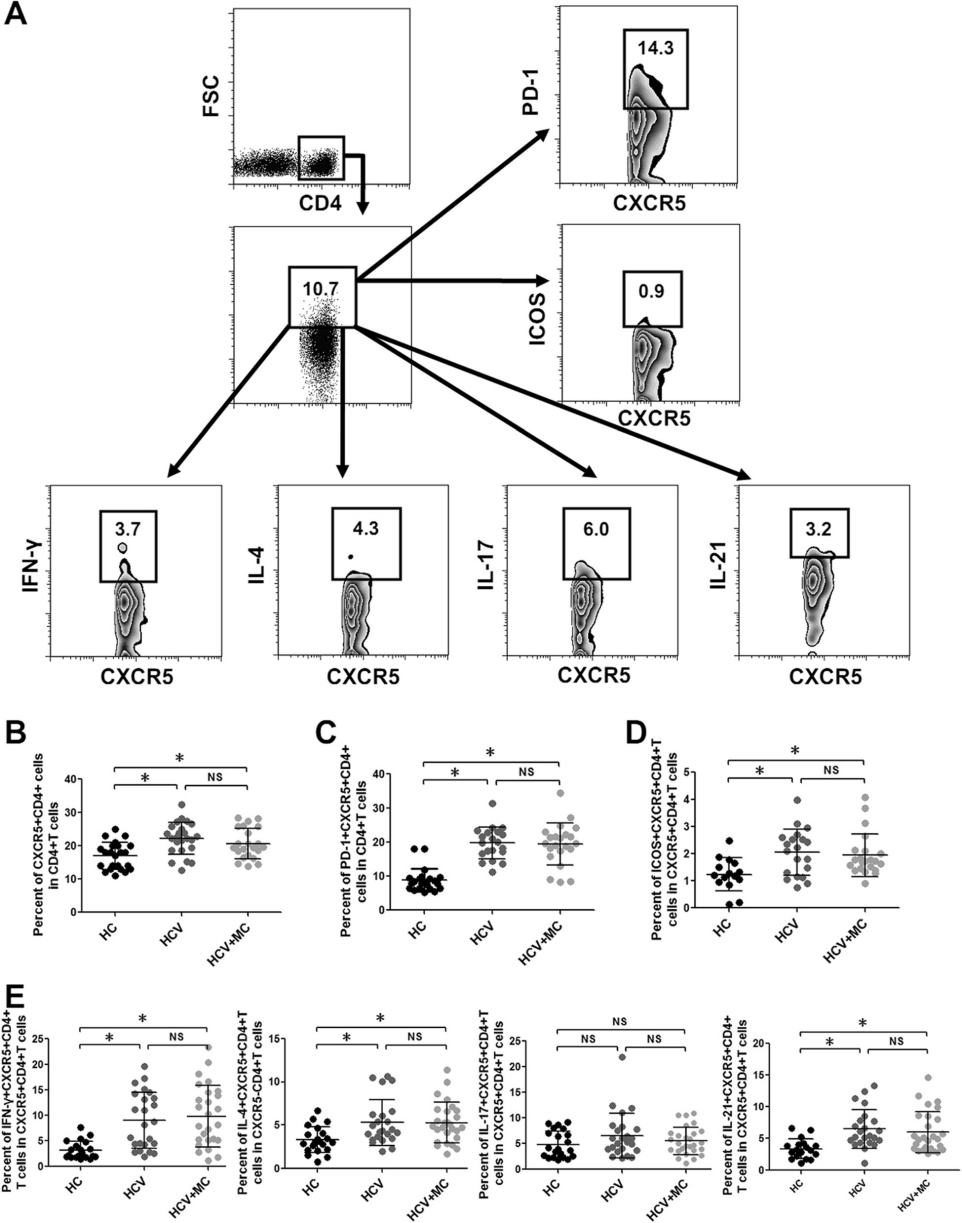
Frequencies of circulating Tfh cell subsets in CHC patients and HCV-related asymptomatic MC patients. **a** The gating strategy for circulating Tfh cell subsets by flow cytometric analysis. **b** Frequency of circulating Tfh cells in CD4 + T cells. **c** Frequency of PD-1+ Tfh cells in CD4+ T cells. **d** Frequency of ICOS+ Tfh cells in total CD4+ T cells. **e** Frequencies of Tfh1 (IFN-γ + Tfh) cells, Tfh2 (IL-4+ Tfh) cells, Tfh 17 (IL-17+ Tfh) cells, and IL-21+ Tfh in total Tfh cells. *p <0.05

Tfh cells (CXCR5 + CD4+ T cells), a major subset of effector CD4 + T cells, are associated with the production of IL-21 and are responsible for the activation and differentiation of B cells [29]. In peripheral blood, circulating Tfh cells were characterized by CXCR5 with ICOS or PD-1, and could be divided into different subsets based on the expression of IFN-γ, IL-4 and IL-17 [30]. Previous studies showed that Tfh cells were associated with HCV infection and autoimmune diseases [28, 31, 32]. Whether Tfh cells are related to type III asymptomatic MC in HCV infection is well understood. We examined the frequencies of different Tfh cell subsets in the three patient groups. As shown in Fig. 3b-d, the frequencies of CXCR5 + CD4+ T cells, PD-1 + CXCR5 + CD4+ T cells and ICOS + CXCR5 + CD4+ T cells in CHC patients and HCV-related MC patients were significantly higher than in HCs, although the frequencies of these Tfh cell subsets were not statistically different in CHC patients and HCV-related MC patients. We further measured different Tfh subsets including IFN-γ + Tfh cells, IL-4+ Tfh cells, IL-17+ Tfh cells, and IL-21 + Tfh cells (Fig. 3e). The results showed that IFN-γ + Tfh cells, IL-4+ Tfh cells, and IL-21+ Tfh cells were associated with HCV infection but not MC. In addition, the frequencies of IL-17+ Tfh cells were comparable in the three groups. Taken together, these results suggest that Tfh cell subsets are associated with HCV but are not involved in type III asymptomatic MC with HCV infection.

### Increased expressions of IFN-γ, IL-17, IL-21, and IL-22 are associated with HCV infection but not type III asymptomatic MC

**Fig. 4:**
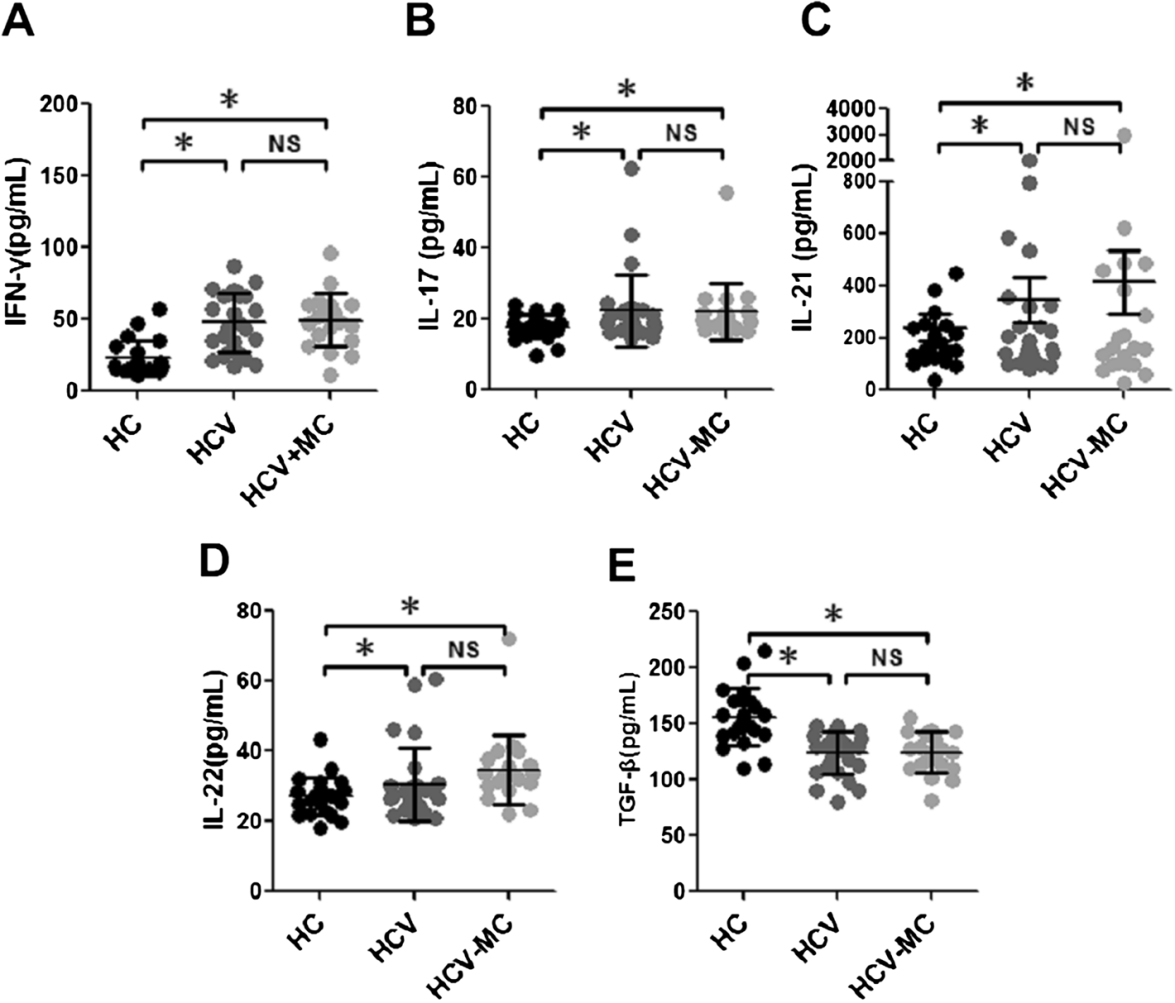
Expressions of serum cytokines in CHC patients and HCV-related asymptomatic MC patients. **a**, **b**, **c**, **d** and **e** show the expression of serum IFN-γ, IL-17, IL-21, IL-22, and TGF-β detected by ELISA. *p <0.05

We next detected the cytokines IFN-γ, IL-4, IL-17, IL-21, IL-22, and TGF-β, which are associated with immune responses of Th1 cells, Th2 cells, Th17 cells, Tfh cells, Th22 cells, and Treg cells. As shown in Fig. 4a-d, the expressions of IFN-γ, IL-17, IL-21, and IL-22 were higher in CHC patients and HCV-related MC patients than in HCs, although this was not significant between CHC patients and HCV-related MC patients. Compared with HCs, the expression of TGF-β was lower in CHC patients and HCV-related MC patients, but was not significantly different between CHC patients and MC patients with HCV infection (Fig. 4e). Because the expressions of IL-4 in most subjects from the three groups were below the detection limit of the enzyme-linked immunosorbent assay (ELISA) kit (15.6 pg/mL), and we could not distinguish differences in IL-4 expression levels (data not shown). In summary, these results suggested that abnormalities of IFN-γ, IL-17, IL-21, IL-22, and TGF-β production were related to HCV infection but not associated with type III asymptomatic MC.

### Frequency of Th1 cells is positively correlated with the frequency of activated memory B cells in HCV-related asymptomatic MC

**Fig. 5:**
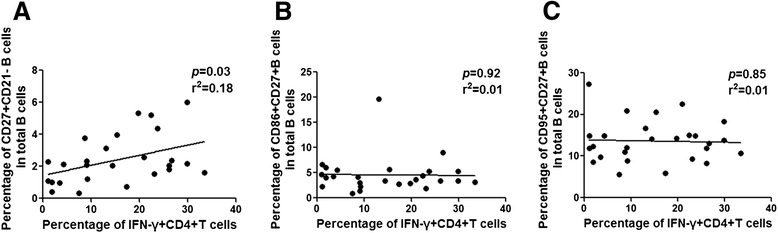
Relationship between frequency of Th1 cells and activated memory B cells in HCV-related asymptomatic MC patients. The relationship of **a** IFN-γ + CD4+ T cells with CD27 + CD21- B cells; **b** IFN-γ + CD4+ T cells with CD86 + CD27+ B cells; and **c** IFN-γ + CD4+ T cells with CD95 + CD27+ B cells

Next we analyzed the relationship of the frequencies of different subsets of CD4+ T cells with B cell subsets in HCV-related asymptomatic MC. The frequency of Th1 cells was positively related to the percentage of activated memory B cells in MC patients with HCV infection (Fig. 5a). However, there were no statistically significant associations of Th1 cells with CD86 + CD27+ B cells or CD95 + CD27+ B cells in HCV-related MC patients (Fig. 5b-c). Together, the results indicated that Th1 cells were associated with the activation of memory B cells, but were not related to the expression of activation markers CD86 and CD95 in asymptomatic MC with HCV infection.

### Frequency of Th1 cells, activated memory B cells are associated with HCV RNA in type III MC

**Fig. 6:**
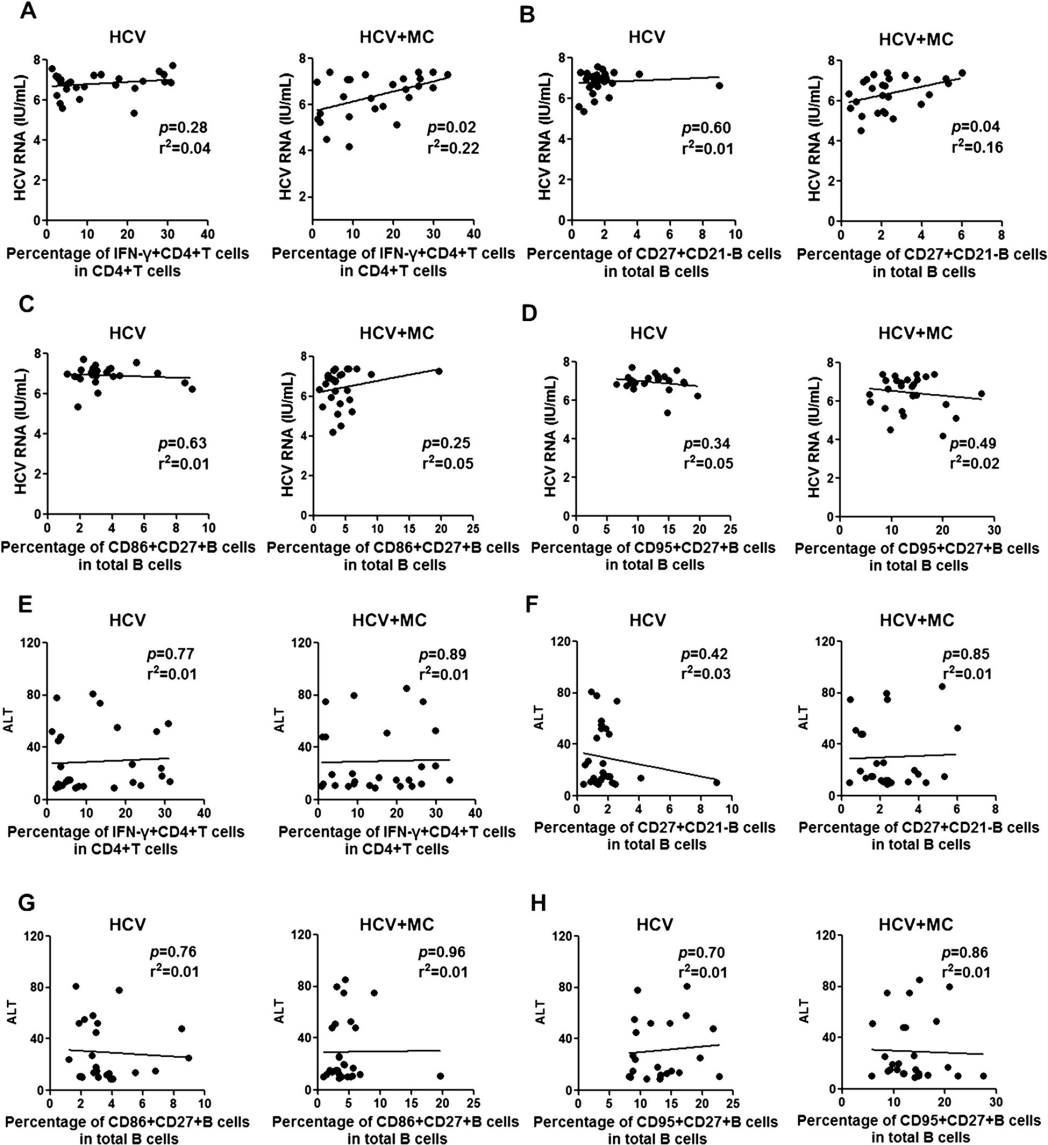
Relationships between frequencies of Th1 cells and activated memory B cells with HCV RNA in CHC patients and HCV-related asymptomatic MC patients. **a** Relationship of Th1 cells with HCV RNA in CHC patients and MC patients with HCV infection. **b** Relationship of CD27 + CD21- B cells with HCV RNA in CHC patients and HCV-related MC patients. **c** Relationship of CD86 + CD27+ B cells with HCV RNA in CHC patients and MC patients with HCV infection. **d** Relationship of CD95 + CD27+ B cells with HCV RNA in CHC patients and HCV-related MC patients. **e** Relationship of Th1 cells with ALT in CHC patients and MC patients with HCV infection. **f** Relationship of CD27 + CD21- B cells with ALT in CHC patients and HCV-related MC patients. **g** Relationship of CD86 + CD27+ B cells with ALT in CHC patients and MC patients with HCV infection. **h** Relationship of CD95 + CD27+ B cells with ALT in CHC patients and HCV-related MC patients

We further measured the relationship of Th1 cells and activated memory B cells with clinical parameters in HCV-related asymptomatic MC patients. We found the frequencies of Th1 cells and activated memory B cells were positively related to HCV RNA in MC patients with HCV infection (Fig. 6a-b). In HCV-related MC patients, the frequencies of CD86 + CD27+ B cells and CD95 + CD27+ B cells were not significantly associated with HCV RNA (Fig. 6c-d). In CHC patients without MC, we failed to detect significant associations of Th1 cells, activated memory B cells, CD86 + CD27+ B cells and CD95 + CD27+ B cells with HCV RNA. We also tested the relationship of alanine transaminase (ALT), a marker of inflammatory necrosis in the liver, with the frequencies of Th1 cells and activated memory B cells in CHC patients and HCV-related asymptomatic MC patients, but the results showed no significant association (Fig. 6e-h). In summary, these results suggested that HCV may have a significant influence on the presence of abnormal Th1 cells and activated memory B cells in type III asymptomatic MC.

## Discussion

Although MC in HCV infection is associated with abnormal immune responses mediated by T cells and B cells, the relationships of different subsets of CD4+ T cells and B cells with type III asymptomatic MC in HCV infection is not well explored. We found increased frequencies of Th1 cells and activated memory B cells in HCV-related type III asymptomatic MC. In addition, the frequency of Th1 cells was positively related to activated memory B cells in HCV-related MC. Moreover, the two target cell subsets were associated with HCV RNA in MC patients with HCV infection.

MC is considered an autoimmune disease, and immune disorders mediated by T cells have been investigated by different research groups. Although reports from Cacoub et al. and Sansonno et al. showed no significant difference in the frequency and activation of T cells in CHC patients and MC patients with HCV infection [33, 34], it is still unknown whether Th cell subsets are involved in the pathogenesis of HCV-related type III asymptomatic MC. In this study, we investigated the relationship of Th cell subsets with HCV-related type III MC without cryoglobulinemic symptoms, and found that Th2 cells, Th17 cells, Th22 cells and IL-21 + CD4+ T cells, were only associated with HCV infection but not MC. A higher frequency of Th1 cells was observed in HCV-related MC patients but not CHC patients in comparison to HCs, implying that immune responses mediated by Th1 cells might have an important role in the progression of HCV-related MC. Previous studies showed that Treg cells were increased in CHC patients [25], while Boyer et al. observed a deficiency of CD4 + CD25+ Treg cells in HCV-related MC patients with active vasculitis compared with CHC patients without MC and HCs [15]. In this study, we found a higher frequency of Treg cells in CHC patients, while a reduced the frequency of Treg cells was observed in MC. In addition, we did not observe a deficiency of Treg cells in HCV-related MC patients without cryoglobulinemic symptoms compared to HCs. The difference between the study by Boyer et al. and our results might be due to the presence or absence of cryoglobulinemic symptoms, which might enhance the inhibition of Treg cells in HCV-related MC patients.

Abnormalities of different subsets of B cells, including immature B cells, tissue-like B cells and memory B cells have been described in MC [17, 18, 20, 21], in studies largely focused on type II MC with cryoglobulinemic symptoms in HCV infection. We investigated different B cell subsets in HCV-related type III asymptomatic MC, and found that the frequency of memory B cells was reduced, while the percentage of tissue-like B cells was increased in HCV infection. These results are consistent with previous studies [35–37]. In the current study, only activated memory B cells with increased expression of activation markers CD86 and CD95 were associated with HCV-related type III asymptomatic MC. Although the features and functions of activated memory B cells, specifically IgM+ memory B cells, has been elucidated in type II MC with HCV infection [17, 18], their exact function in MC patients is still unclear. The composition of cryoglobulins in type III MC is polyclonal IgG and polyclonal IgM, and IgG from memory B cells with class switching [38]. Thus, we hypothesized that activated memory B cells might be a main source of polyclonal IgG in cryoglobulins in type III MC patients with HCV infection.

Considering Tfh cells are responsible for the activation and differentiation of B cells [29], and are involved in HCV infection and autoimmune diseases [28, 31, 32, 39], we speculated that abnormal memory B cell activation may be associated with Tfh cells in HCV-related MC. Unexpectedly, we found higher frequencies of Tfh cell subsets in CHC patients and HCV-related MC patients, and Tfh cells were not related to type III asymptomatic MC. *In vivo* activation of B cells is dependent on BCR stimulation, coupled with help from T cells via coupling of CD40L with CD40 on B cells and cytokine secretion by T cells, or relies on endogenous TLR ligand stimulation [40]. Studies by Charles et al. and Terrier et al. showed that activated memory B cells were anergic to BCR- and CD40-mediated stimulation but not TLR9 triggering in HCV-related MC patients [18, 41], implying endogenous TLR ligands but not Tfh cells were responsible for the aberrant activation of memory B cells in HCV-related MC.

Because the diverse functions of Th cells are mainly determined by the cytokines they produce, we investigated cytokines associated with Th1 cells, Th2 cells, Th17 cells, Tfh cells, Th22 cells, and Treg cells in the three patient groups. We found that the expression of IL-17, IL-21, and IL-22 was increased in CHC patients and HCV-related MC patients, consistent with the frequencies of Th17 cells, Tfh cells and Th22 cells in these patients. We also found the expression of IFN-γ was higher in both CHC patients and HCV-related MC patients, but it was not related to the frequency of Th1 cells in these patients compared to HCs. Furthermore, the expression of TGF-β was lower in CHC patients and HCV-related MC patients, which was also not related to the frequencies of Treg cells in these patients. However, IFN-γ and TGF-β are not only produced by T cells, but can also be secreted by many different types of cells such as professional antigen-presenting cells [42, 43]; thus, IFN-γ and TGF-β from other type cells might have a significant effect on the concentration of serum IFN-γ and TGF-β in CHC patients. Although the prevalence of IFN-γ and other cytokines associated with Th1 immune responses have been observed in CHC patients with MC, other studies mainly focused on MC with cryoglobulinemic symptoms. Our results suggested that type III asymptomatic MC is only associated with a higher frequency of Th1 cells but was not related to IFN-γ in HCV infection. To better understand the effect of cryoglobulinemic symptoms on the expression of Th1 cells, IFN-γ and cytokines associated with Th1 immune responses in HCV-related MC patients should be investigated further by enrolling CHC patients with symptomatic MC in future studies. Our results are consistent with Zhang et al., who found the expression of TGF-β was lower in CHC patients [44], although other studies detected no difference or a higher expression of serum TGF-β levels in CHC patients compared to HCs [45, 46]. This suggested that the divergent genetic background of the study population might have an effect on serum TGF-β levels in HCV infection. MC inhibits the expression of Treg cells, but the exact mechanism is still unknown. TGF-β has critical roles in the differentiation and function of Treg cells [47]; however, a relationship between TGF-β and Treg cells in HCV-related MC patients is not well understood. In the study, we did not find a similar change of TGF-β expression with frequency of Treg cells in HCV-related MC patients compared to CHC patients, implying that MC might induce the reduction and dysfunction of Treg cells in a non-TGF-β-dependent manner.

We next evaluated the relationships among Th1 cells, activated memory B cells, and clinical parameters in HCV-related asymptomatic MC. The frequency of Th1 cells was associated with activated memory B cells in MC patients with HCV infection, suggesting the immune response mediated by Th1 cells might contribute to memory B cell activation in HCV-related asymptomatic MC. In addition, the percentages of Th1 cells and activated memory B cells were related to HCV RNA but not ALT levels in HCV-related MC patients. In CHC patients without MC, the relationship of Th1 cells and activated memory B cells with HCV RNA load was not statistically significant. In chronic HCV infection, changes in Th1 cells and activated memory B cells may gradually increase to levels that contribute to the development of MC disorder and until the stage where type III MC develops. Therefore, the effect of virus infection on Th1 cells and activated memory B cells should be determined.

## Conclusion

This study highlights that a higher frequency of Th1 cells is associated with memory B cell activation in HCV-related type III MC without cryoglobulinemic symptoms. HCV RNA load is closely associated with Th1 cells and activated memory B cells in asymptomatic MC patients with HCV infection. Although a small sample number of patients is tested, our observations indicate that alterations in the frequency of Th1 cells and activated memory B cells might play an important role in the development of type III asymptomatic MC with HCV infection. Th1 cells and activated memory B cells in HCV-infected type III asymptomatic MC patients might also be used as biomarkers for clinical monitoring and disease management. To understand the complex function of Th cells and B cells in MC with HCV infection, further investigations are warranted, by using a larger population and enrolling HCV-related MC patients with cryoglobulinemic syndrome in our future studies.

## Methods

### Patients and controls

Twenty-six CHC patients with type III MC and 28 CHC patients without MC, and 23 HCs were enrolled in the study (Table 1). All CHC patients were infected with genotype 1b HCV. Cryoglobulinemia was defined as the presence of circulating cryoglobulins [48], and the type of cryoglobulins was assessed by western blot assay. Clinical manifestations of cryoglobulinemic syndrome such as vessel vasculitis, glomerulo-nephritis, and neuropathy were not observed in these MC subjects. Liver fibrosis was evaluated by liver stiffness measurement (FibroScan®, Echosens, Paris, France) [49], and none had severe fibrosis or cirrhosis. All subjects were negative for hepatitis B virus or human immunodeficiency virus infection. This study was approved by the ethics committee of Peking University People’s Hospital and written informed consent was obtained from all subjects.

### Clinical tests and peripheral blood mononuclear cell (PBMC) preparation

HCV antibodies, HCV RNA, and HCV genotypes were tested as described previously [49, 50]. PBMCs were isolated by density gradient centrifugation with Ficoll-Paque Plus (GE Health Bio-science, AB, Sweden) as described by Guo et al. [51]. PBMCs were stored in liquid nitrogen until ready for analysis.

### Detection and characterization of cryoglobulins

To detect cryoglobulins, blood was drawn into prewarmed vacutainer tubes and maintained at 37 °C until the serum was separated. Then serum was incubated at 4 °C for one week and then examined daily for the presence of cryoprecipitate. After the serum containing cryoprecipitate was spun in a centrifuge for 15 min at 4 °C, the supernatant was removed, and the pellet was vortexed in ice-cold PBS three times. The sample was then resuspended in 1 mL PBS and re-dissolved by incubation at 37 °C for 1 h. To determine the type of cryoglobulins, western blot was performed, following an established protocol, with antibodies for IgM, IgG, IgA, Lambda and Kappa chains (SouthernBiotech, Birmingham, USA) [52].

### Flow cytometric analysis

Phenotypic analyses of T cells and B cells were performed with anti-human monoclonal antibodies (mAbs): anti-human CD3-PerCP, CD4-FITC, CD19-PerCP and CD21-APC were from BD Biosciences (San Jose, CA, USA). CXCR5-APC, ICOS-PE, PD-1-PE, IFN-γ-PE, IL-4-PE, IL-17-PE, IL-21-PE, IL-22-PE, CD27-FITC, CD86-PE, CD95-PE, CD25-APC, and Foxp3-PE antibodies were obtained from eBiosciences (San Diego, CA, USA). Cells were analyzed by flow cytometry (BD FACSCalibur, San Jose, CA) and data was analyzed by FlowJo software (Tree Star, San Carlos, CA).

### Intracellular cytokine staining

PBMCs were stimulated with 50 ng/mL of phorbol-12-myristate-13-acetate (PMA) and 1 μg/mL of ionomycin (Sigma-Aldrich, St. Louis, USA), in the presence of 1 μl/ml GolgiStop™ (BD Biosciences) at room temperature for 4 h. After washing twice with PBS containing 0.5 % fetal bovine serum, the cells were stained with CD3-PercP, CD4-FITC with or without CXCR5-APC at room temperature in the dark for 20 min. Cells were fixed and permeabilized with Fixation/Permeabilization solution (BD Biosciences) at 4 °C for 20 min. After washing two times in 1× BD Perm/Wash™ buffer, the cells were incubated at room temperature for 20 min with IFN-γ-PE, IL-4-PE, IL-17A-PE, IL-21-PE, and IL-22-PE antibodies or isotype controls. For staining of Foxp3 in CD4 + CD25+ T cells sorted from PBMCs, Foxp3/Transcription Factor Staining Buffer Set (eBioscience) was used, and detection followed the manufacturer’s instructions.

### ELISA

The serum concentration of cytokines IFN-γ, IL-4, IL-17, IL-22, and TGF-β were detected by human ELISA kit (UCallM Biotech Co., Ltd, Wuxi, China). The detection range of IFN-γ, IL-4, IL-17, and IL-22 was from 15.6 to 1000 pg/mL. The detection range of TGF-β was from 31.3 to 2000 pg/mL. Human IL-21 ELISA kit was from eBiosciences, and the detectable concentration was 78 to 5000 pg/mL. The detection of each cytokine was in accordance with the manufacturer’s instructions.

### Statistical analysis

Statistical analysis was by Student’s *t-*test, ANOVA or Mann–Whitney *U*-test according to data distribution using version 16.0 SPSS software (SPSS Inc. Chicago, IL, USA). The data are presented as the mean ± standard deviation (SD). Analysis of correlation between variables was performed by linear regression analysis with Prism Version 5 software (GraphPad). A *p* value <0.05 was considered statistically significant.

## Abbreviations

PBMCs: Peripheral blood mononuclear cells
CHC: Chronic hepatitis C
RF: Rheumatoid factor
Th: T helper
HCs: Health controls
HCV: Hepatitis C virus
MC: Mixed cryoglobulinemia
Treg: Immunoregulatory T
Tfh: Follicular helper T cells
